# The orthopaedic trauma literature: an evaluation of statistically significant findings in orthopaedic trauma randomized trials

**DOI:** 10.1186/1471-2474-9-14

**Published:** 2008-01-29

**Authors:** Jinsil Sung, Judith Siegel, Paul Tornetta, Mohit Bhandari

**Affiliations:** 1 Department of Orthopaedic Surgery, Boston University, 818 Harrison Ave, Dawling 2 North, Boston Massachusetts, 02118-2393, USA; 2 Department of Surgery, McMaster University, 293 Wellington Street N, Suite 110, Hamilton, Ontario, L8L 8E7, Canada

## Abstract

**Background:**

Evidence-based medicine posits that health care research is founded upon clinically important differences in patient centered outcomes. Statistically significant differences between two treatments may not necessarily reflect a clinically important difference. We aimed to quantify the sample sizes and magnitude of treatment effects in a review of orthopaedic randomized trials with statistically significant findings.

**Methods:**

We conducted a comprehensive search (PubMed, Cochrane) for all randomized controlled trials between 1/1/95 to 12/31/04. Eligible studies include those that focused upon orthopaedic trauma. Baseline characteristics and treatment effects were abstracted by two reviewers. Briefly, for continuous outcome measures (ie functional scores), we calculated effect sizes (mean difference/standard deviation). Dichotomous variables (ie infection, nonunion) were summarized as absolute risk differences and relative risk reductions (RRR). Effect sizes >0.80 and RRRs>50% were defined as large effects.

Using regression analysis we examined the association between the total number of outcome events and treatment effect (dichotomous outcomes).

**Results:**

Our search yielded 433 randomized controlled trials (RCTs), of which 76 RCTs with statistically significant findings on 184 outcomes (122 continuous/62 dichotomous outcomes) met study eligibility criteria. The mean effect size across studies with continuous outcome variables was 1.7 (95% confidence interval: 1.43–1.97). For dichotomous outcomes, the mean risk difference was 30% (95%confidence interval:24%–36%) and the mean relative risk reduction was 61% (95% confidence interval: 55%–66%; range: 0%–97%). Fewer numbers of total outcome events in studies was strongly correlated with increasing magnitude of the treatment effect (Pearson's R = -0.70, p < 0.01). When adjusted for sample size, the number of outcome events revealed an independent association with the size of the treatment effect (Odds ratio = 50, 95% confidence interval: 3.0–1000, p = 0.006).

**Conclusion:**

Our review suggests that statistically significant results in orthopaedic trials have the following implications-1) On average large risk reductions are reported 2) Large treatment effects (>50% relative risk reduction) are correlated with few number of total outcome events. Readers should interpret the results of such small trials with these issues in mind.

## Background

Evidence-based medicine posits that health care research is founded upon clinically important differences in patient centered outcomes. Randomized trials continue to represent the reference standard for the comparison of surgical interventions [1-4]. Although fundamentally the most important for guiding clinical practice, few randomized trials are conducted in orthopaedic surgery. Current estimates suggest that less than 5% of the orthopaedic literature represent randomized trials [5-7]. Nevertheless, the impact of randomized trials, especially those with statistically significant findings, is large [8].

Statistically significant differences between two treatments may not necessarily reflect a clinically important difference. Although it is well known that orthopaedic studies with small sample sizes risk underpowered false negative conclusions (Beta-errors) [6,9,10], statistically significant findings in small trials can occur at the consequence of very large differences between treatments (treatment effect). It is not uncommon for randomized trials to report relative risk reductions larger than 50% when comparing one treatment with another [11-13].

Devereaux and colleagues caution the interpretation of small trials in cardiology [14]. For example, the peri-operative beta-blocker evidence suggests large treatment effects (i.e., relative risk reductions >75%) but these results are inconsistent with beneficial cardiovascular therapies established in trials with 10,000s of patients, which generally demonstrate moderate relative risk reductions in the order of 15 to 35% [14-16].

Our study had 2 objectives: 1) To determine the magnitude of treatment effects in a sample of orthopaedic randomized trials with statistically significant results and 2) to examine the association between the number of outcome events (a measure of study sample size) and the size of the treatment effect. We conducted a systematic review to identify randomized trials in orthopaedic trauma with the following hypotheses: 1) statistically significant studies would not always report large treatment effects and 2) studies with smaller sample sizes (and few outcome events) would be more likely to report larger treatment effects than those with larger sample sizes.

## Methods

### Eligibility Criteria

We included studies which met the following eligibility criteria: 1) published studies, 2) described as randomized trial, 3) involve the care of adult patients with fractures, either operative or conservative, 4) published in English and 5) contain sufficient outcomes information to calculate treatment effects for both dichotomous and continuous outcome measures. Our decision to focus upon trauma randomized trials was based upon two factors: 1) allowing comparison with previous studies evaluating this population of trials, and 2) practicality of limiting the number of trials to a sufficiently manageable number to optimize the efficiency of study completion and research resource utilization within our Departments.

### Study Identification

We conducted a comprehensive search (PubMed, Cochrane database) for all randomized controlled trials between January 1, 1995 and December 31,2004. We used the search terms "randomized controlled trial" and "fracture" and "surgery" with limits (adults 19+ years). The eligibility criteria were applied to potentially eligible study titles by two independent reviewers (JS, MB). One of the two reviewers was trained in health research methodology, while the other was an orthopaedic resident. Abstracts for those eligible study titles were retrieved by one of us. Following a second application of eligibility criteria to abstracts by independent reviewers, complete citations for those potentially eligible studies were retrieved. The methods section of each retrieved citation was reviewed by two of us to ensure all inclusion criteria were met. In addition to Medline searches, two of us performed a search of the NIH PubMed computerized database and one of us conducted a Cochrane Database search. For both searches, we used "fractures" and "randomized trials" as keywords.

Additional strategies to identify relevant citations included: 1) hand searches of the table of contents over the past 5 years of the Journal of Orthopaedic Trauma, Journal of Trauma, Clinical Orthopaedics and Related Research and Acta Orthopaedica Scandinavica, 2) review of the reference lists of eligible (included) studies to identify other potentially eligible studies, and 3) content experts' (traumatologist) review of the list of eligible studies to identify any missing studies.

### Characteristics of Eligible Studies

Two reviewers independently abstracted general characteristics of each eligible study. These included, first author (surgeon/non-surgeon), geographic location, category of intervention, body region of focus (upper extremity, lower extremity, spine), number of participating centres, and funding (yes/no).

### Determination of Treatment Effects and Outcome Events

For dichotomous outcome measures (ie re-operation, infection), we calculated relative risks as percent (%) re-operation intervention group divided by percent (%) re-operation in comparison group. For ease of interpretation we converted relative risks to relative risk reductions [(1-Relative Risk) × 100]. We also calculated absolute risk differences. A relative risk reduction of 50% was interpreted as Experiment treatment reduced the risk of an adverse outcome event by 50% compared to a control (comparison treatment).

For continuous outcome measures (ie functional scores) we calculated an effect size as described by Cohen [17]. 'Effect Size' is simply a way of quantifying the effectiveness of a particular intervention, relative to some comparison. It is easy to calculate, readily understood and can be applied to any measured outcome in surgical trials. It is the standardized mean difference between the two groups. We used the following formula for its calculation:

$$EffectSize=\frac{\left[Meanofexperimentalgroup]-[MeanofComparisongroup\right]}{PooledStandardDeviation}$$

For example, an effect size of 0.8 means that the score of the average person in the experimental group exceeds the scores of 79% of the comparison group. Cohen describes an effect size of 0.2 as 'small' and gives to illustrate it the example that the difference between the heights of 15 year old and 16 year old girls in the US corresponds to an effect of this size. An effect size of 0.5 is described as 'medium' and is 'large enough to be visible to the naked eye'. A 0.5 effect size corresponds to the difference between the heights of 14 year old and 18 year old girls. Cohen describes an effect size of 0.8 as 'grossly perceptible and therefore large' and equates it to the difference between the heights of 13 year old and 18 year old girls [17]. For each study, we documented outcomes measured deemed primary by the study authors. In cases in which primary outcomes were not specified by authors, two of us (trained orthopaeidic surgeons) made judgments about the key outcomes based upon the study interventions.

### Assessing Reviewer Agreement

Agreement in the application of study eligibility criteria, identification of study outcomes and study results (positive or negative) was quantified with the Kappa statistic with quadratic weighting. The kappa statistic, a measure of the agreement between two or more individuals beyond chance, provided a measure of agreement among reviewers for titles, abstracts and methods sections of potentially relevant. In the context of inter-observer agreement studies, Fleiss and Donner provide persuasive arguments favoring the use of this statistic over other measures of agreement that have been proposed [18,19].

### Data Analysis

We presented descriptive statistics about continuous variables with means, standard deviations and dichotomous variables as proportions. We calculated relative risks and 95% confidence intervals to describe treatment effects and compared relative risks in studies with few and many events with tests for interaction. Logistic regression provided methods for estimating the extent of association between the total number of events (ie, endpoints driving termination) in the trial and the calculated treatment effect. We categorized the number of outcome events as follows: 1) 0–25 events, 2) 26–50 events, 3) 51–75 events, and 4) 76–100 events. We also categorized studies as having a relative risk reduction as follows: 1) <50% and 2)>50%. We expressed any associations using odds ratios (ORs) and associated 95% confidence intervals (CIs). Analyses were performed using SPSS version 13.0. We conducted a correlation analysis of the number of outcome events against the treatment effect with a Pearson's R. We considered P < 0.05 as the level of statistical significance for all comparisons. All tests were two- tailed.

## Results

### Literature Search

**Figure 1:**
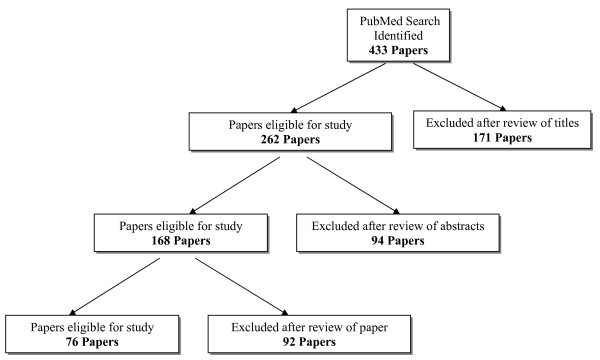
Literature search.

We identified 433 potentially relevant study titles from the MEDLINE database search. (Figure 1). Application of the study eligibility criteria eliminated 171 titles, leaving 262 for further consideration (Table 1). Following complete review of 262 study abstracts, 94 were excluded, leaving 168 papers. In total, 168 studies appeared potentially eligible from study title and abstract alone and full manuscripts were retrieved for a detailed review. Agreement in the application of eligibility criteria to study titles and abstracts was substantial (Kappa = 0.80, 95% confidence interval: 0.74–0.86). Application of the eligibility criteria to the complete manuscripts of 168 trials eliminated 92 studies (Table 1). Thus, 76 papers that met all *apriori *eligibility criteria were included in the analyses (**Appendix**).

**Table 1:** Reasons for Study Exclusion (N = 96 excluded) from Full Manuscript Review

Reason for Exclusion	Number
*I. Reasons for Study Exclusion (N = 171 excluded) from Title Review*	
Medical Interventions	50
Anesthesia Papers	46
OMFS/ENT/Ophthalmology/Dental	22
Rehabilitation Paper	13
DVT Papers	6
Antibiotic Papers	6
Nutrition Papers	4
Miscellaneous	34
*II. Reasons for Study Exclusion (N = 92 excluded) from Full Manuscript Review*	
No data available	36
Non-English Journal	24
Insufficient Data to Calculate Effect Sizes	16
Journal Unobtainable	6
Not Statistically Significant Data	4
Medical Interventions	2
Non trauma Spine	1
Non trauma Sports	1
Rehabilitation Paper	1
Retrospective Paper	1

### Study Characteristics

The 76 eligible trials were published across 9 different journals (Table 2). The majority of studies were published in Journal of Orthopaedic Trauma (21%), JBJS-British (18%), and JBJS-American (13%). Most of the studies were conducted in Europe (58%) followed by North America (32%) (Table 3). Seventy-three (96%) of the first authors were surgeons and the majority of studies were single center initiatives (83%). Funding for the conduct of the trial was reported in 17 studies (22%). A total of 9757 patients were randomized in the 76 trials. Moreover, study sample sizes ranged from 10 to 424 patients (mean = 77 patients, st.dev = 69).

**Table 2:** Literature Search – Journals for 76 Included Articles

**Journal Title**	**# of Articles**
Acta Orthopaedica Scandinavica	8
Journal of Bone and Joint Surgery – British Volume	14
Clinical Orthopaedics and Related Research	9
Injury	5
Journal of Bone and Joint Surgery – American Volume	10
Journal of Orthopaedic Trauma	16
Journal of Trauma	8
Journal of Hand Surgery – British Volume	4
Foot and Ankle International	2

**Total**	**76**

**Table 3:** Characteristics of the Eligible Trials (N = 76 Trials)

Characteristics		No. of Studies (Percent)	JOT	JBJS-Am	JBJS-Br	CORR	Acta Orth. Scand	Journal of Trauma	Injury	Foot and Ankle Internat.	Journal Hand-Br.
1.	First Author:										
	Surgeon:	73/76 (96.0%)	15/16	10/10	13/14	8/9	8/8	8/8	5/5	2/2	4/4
	Non-Surgeon:	3/76 (4%)	1/16	0/10	1/14	1/9	0/8	0/8	0/5	0/2	0/4
	Epidemiologist:	0 (0%)	0/16	0/10	0/14	0/9	0/8	0/8	0/5	0/2	0/4
											
2.	Epidemiology Affiliation:										
	Yes:	16/76 (21.0%)	0/16	2/10	10/14	0/9	1/8	3/8	0/5	0/2	0/4
	No:	60/76 (78.9%)	16/16	8/10	4/14	9/9	7/8	5/8	5/5	2/2	4/4
											
3.	Geographical Location:										
	North America:	24/76 (32%)	9/16	6/10	1/14	5/9	0/8	2/8	0/5	1/2	0/4
	Europe (includes England):	44/76 (58%)	7/16	4/10	10/14	4/9	8/8	2/8	4/5	1/2	4/4
	Australia or New Zealand:	1/76 (1.%)	0/16	0/10	1/14	0/9	0/8	0/8	0/5	0/2	0/4
	Asia:	6/76 (8%)	0/16	0/10	2/14	0/9	0/8	3/8	1/5	0/2	0/4
	Israel:	1/76 (1%)	0/16	0/10	0/14	0/9	0/8	1/8	0/5	0/2	0/4
											
4.	Category of Intervention:										
	Fracture Treatment:	70/76 (92.1%)	16/16	9/10	12/14	8/9	7/8	7/8	5/5	2/2	4/4
	Other Treatment:	5/76 (6.6%)	0/16	1/10	1/14	1/9	1/8	1/8	0/5	0/2	0/4
	Vascular Treatment:	1/76 (1.3%)	0/16	0/10	1/14	0/9	0/8	0/8	0/5	0/2	0/4
											
5.	Region of Body:										
	Upper Extremities:	16/76 (21.0%)	2/16	5/10	2/14	0/9	0/8	1/8	2/5	0/2	4/4
	Lower Extremity Long Bones:	23/76 (30.3%)	9/16	1/10	4/14	2/9	1/8	5/8	1/5	0/2	0/4
	Spine:	2/76 (2.6%)	0/16	1/10	0/14	1/9	0/8	0/8	0/5	0/2	0/4
	Hip:	28/76 (36.8%)	4/16	2/10	6/14	6/9	6/8	2/8	2/5	0/2	0/4
	Foot and Ankle	4/76 (5.3%)	0/16	1/10	0/14	0/9	1/8	0/8	0/5	2/2	0/4
	Knee:	2/76 (2.6%)	1/16	0/10	1/14	0/9	0/8	0/8	0/5	0/2	0/4
	Upper and Lower Extremity:	1/76 (1.3%)	0/16	0/10	1/14	0/9	0/8	0/8	0/5	0/2	0/4
											
6.	Single or Multicentred:										
	Single:	63/76 (83%)	15/16	6/10	13/14	6/9	5/8	8/8	5/5	2/2	3/4
	Multicentred:	13/76 (17%)	1/16	4/10	1/14	3/9	3/8	0/8	0/5	0/2	1/4
											
7.	Funding:										
	Yes:	17/76 (22%)	2/16	4/10	4/14	2/9	4/8	0/8	0/5	0/2	1/4
	No:	27/76 (36%)	10/16	4/10	10/14	1/9	0/8	0/8	1/5	0/2	1/4
	Unknown:	32/76 (42%)	4/16	2/10	0/14	6/9	4/8	8/8	4/5	2/2	2/4

* JBJS = Journal of Bone and Joint Surgery, JOT = Journal of Orthopaedic Trauma; CORR = Clinical Orthopaedics and Related Research

### Treatment Effects

We identified 62 dichotomous outcomes across our sample of 76 studies (Table 4). The mean sample size in studies that reported dichotomous outcomes was 81.2 ± 61.4 patients (median 55 patients) with a minimum 14 patients to a maximum 250 patients. For dichotomous outcomes, the mean risk difference was 30% (95%confidence interval:24%–36%; range 2%–138%). The treatment effects (relative risks) averaged 0.39 (95% confidence interval: 0.33–0.45; range: 0.03–1.0). This translated to a mean observed relative risk reduction of 61% (95% confidence interval: 55%–66%; range: 0%–97%). Thus, the average study reported large reductions (>50% relative risk reduction) in the risk of an adverse outcome event versus a comparative treatment; however, almost 1 in 2 study outcomes (47%) had RRRs<50%, and over 1 in 5 (23%) had RRRs<20%.

**Table 4:** Outcome Measures Reported in 76 Trials

**Continuous**
Functional/Clinical Score
OR time/Surgical time/Fluoroscopy time
Blood loss
Grip strength/Power
Time to Union
Range of Motion
Radiographic Results/Scores
Days in hospital
Load to failure

**Dichotomous**

General complications
Reoperation
Failure
Nonunion/Union/Malunion
Pain
Infection
Mortality
Need for transfusion
Segmental Collapse/Avascular Necrosis
Parasthesia
Return to preinjured state
Return to work

We identified 122 continuous outcomes across our sample of 76 studies (Table 4). The mean sample size in studies reporting continuous outcomes was 70 ± 69 patients (median = 50 patients) with a minimum 10 patients and a maximum 424 patients. The absolute mean difference between treatments was 81.2 (95% confidence interval: 49.2–113.2). The effect size averaged 1.7 (95% confidence interval: 1.43–1.97). Eighty four outcomes reported (69%) large effect sizes (ES>0.80).

### Association between Treatment Effect and the Number of Outcome Events

**Figure 2:**
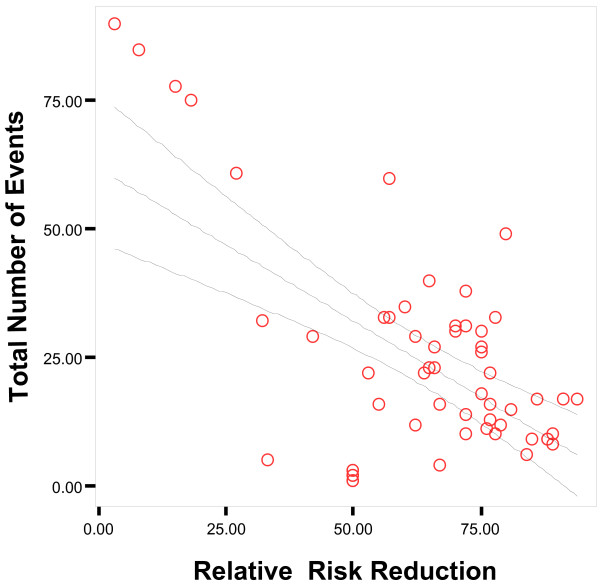
**Scatter plot of Total Number of Events Versus Relative Risk Reduction**. Regression lines with 95% confidence interval is presented. Total Number of Events = 62 – 0.59(Relative Risk Reduction).

**Figure 3:**
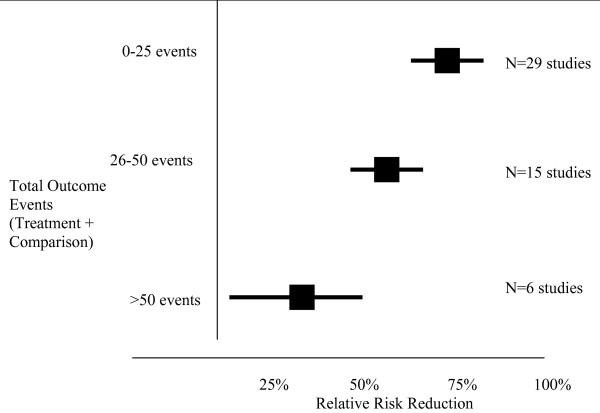
Total Number of Events Versus Treatment Effect.

The mean number of outcome events (Treatment group + Comparison Group) across studies with dichotomous outcomes was 24 ± 21 (median = 17). The total number of outcome events ranged from 1 event to 90 events. Fewer numbers of total outcome events in studies was strongly correlated with increasing magnitude of the treatment effect (Pearson's R = -0.70, p < 0.01) (Figure 2). The relative risk reduction decreased as the number of outcome events increased from 0–25 events to 75–100 events (Rel. Risk Red = 73% vs 16%, respectively, P < 0.01) (Table 5) (Figure 3). In the logistic regression analysis, fewer than 50 outcome events was significantly associated with a relative risk reduction greater than 50% (Odds ratio = 21, 95% confidence interval: 2.1–200, p = 0.0–09). When adjusted for sample size, the number of outcome events continued to show independent association with the size of the treatment effect (Odds ratio = 50, 95% confidence interval: 3.0–1000, p = 0.006). The number of outcome events explained 32% of the variance in the regression model. In the 6 studies with greater than 50 total outcome events, 5 studies had modest to small relative risk reductions range (3%–33%).

**Table 5:** Treatment Effects and Outcome Events for Dichotomous Variables

Number Outcome Events	No. Studies	Relative Risk* Reduction	95% confidence Interval	P Value
0–25	29	73%	67%–79%	
				P = 0.007
25–50	15	59%	50%–67%	
				P = 0.005
51–75	4	33%	15%–50%	
				P = 0.20
75–100	2	16%	0.01%–41%	

* Represented as the mean relative risk reduction across studies

## Discussion

Our review of trials with statistically significant findings in orthopaedic trauma suggests the following: 1) trials have small sample sizes 2) average treatment effects are large and 3) large treatment effects (>50% relative risk reduction) correlated with fewer number of total outcome events. Readers should interpret the results of such small studies with few events with caution.

### Strengths and Limitations

Our study is strengthened by a comprehensive literature review including hand searches of major orthopaedic journal, careful study methodology and duplicate data abstraction. Our search strategy, although comprehensive may have missed studies related to fracture care due to errors in study indexing in Medline or those articles not indexed in PubMED. Our decision to identify only trials relating to orthopaedic trauma for pragmatic reasons limits our generalizability beyond this subspecialty. However, it remains plausible that the associations we observed are consistent throughout the orthopaedic literature in both English and Non-English trials. The lack of reporting of sufficient data to calculate treatment effect size for those studies with continuous outcome variables was another limitation. Fifty-two studies were excluded for this reason. We realize that excluding so many studies is a limitation. However, review of these studies suggests that they were similar in sample size, geographic location, funding status and number of centers to our included studies.

### Relevant Literature Review

Whereas statistical significance means the likelihood that the difference found between groups could have occurred by chance alone, effect-sizes provide an estimate of the size of the treatment effect. Effect sizes are important because they facilitate the comparison of treatment effects across different studies. In most clinical trials, a result is statistically significant if the difference between groups could have occurred by chance alone in less than 1 time in 20 (i.e. less than 5% probability, p < 0.05). A trivial difference can have a low p value (i.e. much less than p < 0.05) if the sample size of the study is large. For example, a large trial (N = 7601 patients) comparing the use effects of the angiotensin receptor blocker candesartan on cardiovascular outccomes reported a significant reduction in the development of atrial fibrillation with candesartan versus placebo (p = 0.048); however, the actual difference was 1.19% (6.74% vs 5.55%, respectively) [20].

The findings reported in some biomechanical studies should also be interpreted cautiously. For example, a biomechanical study that compared the compression effect of the 7.0-AO screw and the 6.5 mm Ideal Compression Screw (I.CO.S.) screw in an in vitro subtalar arthodesis model. The authors reported that the AO screw led to a significantly increased mean contact force (p < 0.05); however, this increase was 7.6 N – the clinical significance of which is completely uncertain [21].

Clinical significance is a matter of judgment. However, clinically significant findings imply that the difference between treatment groups are large enough to be important to patients. We can argue that an absolute difference of 1.19% is not a clinically important difference. Alternatively, a study of 50 patients reporting a 20% (10/25, 40% vs 5/25, 20%, p = 0.12) absolute difference in atrial fibrillation rates between candesartan and placebo groups may be more compelling for clinical practice. However, the difference may not reach statistical significance.

There have been no studies in the surgical literature evaluating the association of treatment effect magnitudes and number of outcome events. However, investigators have examined inflated treatment effects in large medical trials stopped early for benefit at an early interim analysis [16]. For example, a trial that aimed to recruit 1000 patients may be terminated early if an interim analysis of 100 patients shows a very large benefit of the treatment over a comparison (ie relative risk reduction >50%). The decision to terminate a trial before it reaches its preplanned sample size is based upon apriori statistical stopping rules (threshold of a p value is reached). If we extrapolate this to orthopaedic surgical trials, we can conceptualize these small trials (mean = 80 patients) with large treatment effects (relative risk reductions of >66%) as trials that were essentially "stopped early". In doing so, the same play of chance positive findings can be extrapolated to this literature. Moreover, implications of stopped early trials can be explored from previous reports in the medical literature.

Several important lessons about early stopping of trials with large treatment effects exist in the literature [15,16]. For example, the preliminary results of the twelfth Medical Research Council acute myeloid leukemia trial ultimately revealed no evidence of a survival advantage for five courses of therapy compared to four courses in a randomized comparison involving 1078 patients (hazard ratio 1.09, 95% confidence interval [CI] 0.87–1.37, p = 0.4) [15]. However, large benefits of the 5 course therapy (53% and 45% reductions in the odds of death) in early interim analyses of fewer patients recruited were fortunately dismissed as "too good to be true" and implausible. The ultimate large trial prevented the wide adoption of a non-beneficial therapy with potential harms of more chemotherapy.

Another poignant example of the pitfalls of small trials and large effects is derived from Cardiology – the case of magnesium in acute myocardial infarction [22,23]. When initial small trials have claimed remarkably large benefits, subsequent trials typically demonstrated much more modest. For example, a meta-analysis of RCTs of magnesium in acute myocardial infarction demonstrated a statistically significant (*P *< 0.001) > 50% reduction in death with approximately 1,500 patients randomized [20]. However, the subsequent RCT of approximately 60,000 patients showed no benefit; in fact there was a trend toward excess mortality with magnesium (*P *= 0.07) [23]. Devereaux and colleagues argue that cardiovascular therapies rarely demonstrate relative risk reductions greater than 35%, because cardiovascular therapies usually only target a limited number of the multitude of pathogenic mechanisms underlying cardiovascular diseases, as is the case with peri-operative beta-blockers [24].

Take, for example, the RCT evaluating the efficacy of bisoprolol (Beta-blocker) in patients with a positive dobutamine echocardiography result and undergoing elective vascular surgery [25]. The trial was stopped when investigators had enrolled 112 patients of the pre-planned 266 patients. The relative risk reduction for the primary outcome (cardiac death or nonfatal myocardial infarction) was 91% (95% CI, 63%–98%). This very large treatment effect is implausible given that the authors anticipated a less beneficial effect of this drug. In fact, a subsequent trial of larger sample size and greater outcome events (496 patients) undergoing vascular surgery that reported no significant effect of Beta-blockers on cardiac death or nonfatal myocardial infarction [26].

Montori and colleagues recently conducted a systematic review to identify randomized trials stopped early for benefit [16]. Of 143 trials stopped early for benefit, the majority (92) were published in high-impact medical journals (New England Medical Journal, Journal of the American Medical Association, Annals of Internal Medicine). On average, trials recruited 63% of the planned sample and stopped after when a median of 66 total outcome events (experimental + control). The median relative risk reduction among truncated trials was 47%. Trials with fewer events yielded greater treatment effects (odds ratio, 28; 95% confidence interval, 11–73).

### Importance of Our Findings

If one accepts that our sample of orthopaedic randomized trials represents small trials with implausibly large benefits, then our findings are interesting and the implications of our study highly relevant and important. The average study in our review had a sample size of 81 patients but reported a large beneficial treatment effect (61% relative risk reduction). Surgeons should consider the plausibility of the magnitude of the treatment effect because chance effects do occur and happen more frequently than many of us realize [15]. Statistical simulation studies have shown that RCTs can overestimate the magnitude of the treatment effect depending on the timing (ie, the fraction of the total planned sample size or expected number of events) of the decision to stop [27].

Indirect evidence for the implausibility of treatment effects reported in small orthopaedic trauma trials with large reported treatment benefits is available. For example, early small randomized trials (sample sizes 48–141) comparing reamed versus non-reamed intramedullary nailing identified relative risk reductions in nonunion with reamed nailing of 54% (but as high as 79%) [27]. If surgeons truly believed that reamed nails could reduce the risk of an important adverse event by over 50%, surely every surgeon would have adopted this relatively simple strategy [28]. However, surveys of surgeons conducted years after the conduct of these trials found that 42% of surgeons continued to use the non-reamed nail [29]. It remains plausible, then, that the surgical community believed that 54% risk reductions were "implausibly" high.

In another example, pooled analyses small randomized trials have reported large reductions in the risk of reoperation (74%) with plates compared to intramedullary nails in the treatment of humeral shaft fractures [30-32]. Again, if practicing surgeons believed these trials, they would abandon the use of nails. This is not so. The continued use of intramedullary nails for humeral shaft fractures suggests, at least in part, possible skepticism with small trials with large treatment effects.

### Recommendations

Authors should cautiously interpret the positive findings of studies when sample sizes of the study are small and the total number of outcome events are few. As the number of outcome events increases, surgeons should have increasingly greater confidence in the reported magnitude of the treatment effect. For example, a trial claiming that reamed intramedullary tibial nails reduce the risk of revision surgery by 50% in 1000 patients with 200 outcome events is less likely influenced by the random chance than a similar study of 100 patients with 20 outcome events.

Wright and Gebhardt call for specialty orthopaedic societies to take action towards the conduct of multicenter randomized trials [33]. Single center initiatives will rarely be sufficient to enroll sufficiently large numbers of patients efficiently.

## Conclusion

Our review suggests that statistically significant results in orthopaedic trials have the following implications-1) On average large risk reductions are reported 2) Large treatment effects (>50% relative risk reduction) are correlated with few number of total outcome events. Readers should interpret the results of such small trials with these issues in mind.

## Appendix: list of 76 included RCTs

Adolfsson L, Lindau T, Arner M: **Acutrak screw fixation versus cast immobilisation for undisplaced scaphoid waist fractures**. *J Hand Surg Br *2001 Jun, **26(3)**:192-5.

Ahrengart L, Tornkvist H, Fornander P, Thorngren KG, Pasanen L, Wahlstrom P, Honkonen S, Lindgren U: **A randomized study of the compression hip screw and Gamma nail in 426 fractures**. *Clin Orthop *2002 Aug, **(401)**:209-22.

Baumgaertner MR, Curtin SL, Lindskog DM: **Intramedullary versus extramedullary fixation for the treatment of intertrochanteric hip fractures**. *Clin Orthop Relat Res *1998 Mar, **(348)**:87-94.

Bednar DA, Raducan V: **External spinal skeletal fixation in the management of back pain**. *Clin Orthop Relat Res *1996 Jan, **(322)**:131-9.

Bond CD, Shin AY, McBride MT, Dao KD: **Percutaneous screw fixation or cast immobilization for nondisplaced scaphoid fractures**. *J Bone Joint Surg Am *2001 Apr, **83-A(4)**:483-8.

Buciuto R, Uhlin B, Hammerby S, Hammer R: **RAB-plate vs Richards CHS plate for unstable trochanteric hip fractures. A randomized study of 233 patients with 1-year follow-up**. *Acta Orthop Scand *1998 Feb, **69(1)**:25-8.

Buckley R, Tough S, McCormack R, Pate G, Leighton R, Petrie D, Galpin R: **Operative compared with nonoperative treatment of displaced intra-articular calcaneal fractures: a prospective, randomized, controlled multicenter trial**. *J Bone Joint Surg Am *2002 Oct, **84-A(10)**:1733-44.

Burd TA, Hughes MS, Anglen JO: **Heterotopic ossification prophylaxis with indomethacin increases the risk of long-bone nonunion**. *J Bone Joint Surg Br *2003 Jul, **85(5)**:700-5.

Braakman M, Oderwald EE, Haentjens MH: **Functional taping of fractures of the 5th metacarpal results in a quicker recovery**. *Injury *1998 Jan, **29(1)**:5-9.

Calder J, Anderson GH, Jagger C, Harper WM, Gregg PJ: **Unipolar or bipolar prosthesis for displaced intracapsular hip fracture in octogenarians: a randomised prospective study**. *J Bone Joint Surg Br *1996 May, **78(3)**:391-4.

Calder SJ, Barnes MR, Harper WM: **Reduction of temperatures generated by the triple reamer within the femoral head**. *Injury *1995 Apr, **26(3)**:183-5.

Canadian Orthopaedic Trauma Society: **Nonunion following intramedullary nailing of the femur with and without reaming. Results of a multicenter randomized clinical trial**. *J Bone Joint Surg Am *2003 Nov, **85-A(11)**:2093-6.

Carpenter JE, Kasman RA, Patel N, Lee ML, Goldstein SA: **Biomechanical evaluation of current patella fracture fixation techniques**. *J Orthop Trauma *1997 Jul, **11(5)**:351-6.

Carragee, EJ: **Single-level posterolateral arthrodesis, with or without posterior decompression, for the treatment of isthmic spondylolisthesis in adults. A prospective, randomized study**. *J Bone Joint Surg Am *1997 Aug, **79(8)**:1175-80.

Cassidy C, Jupiter JB, Cohen M, Delli-Santi M, Fennell C, Leinberry C, Husband J, Ladd A, Seitz WR, Constanz B: **Norian SRS cement compared with conventional fixation in distal radial fractures. A randomized study**. *J Bone Joint Surg Am *2003 Nov, **85-A(11)**:2127-37.

Chan YS, Ueng SW, Wang CJ, Lee SS, Chen CY, Shin CH: **Antibiotic-impregnated autogenic cancellous bone grafting is an effective and safe method for the management of small infected tibial defects: a comparison study**. *J Trauma *2000 Feb, **48(2)**:246-55.

Chapman JR, Henley MB, Agel J, Benca PJ: **Randomized prospective study of humeral shaft fracture fixation: intramedullary nails versus plates**. *J Orthop Trauma *2000 Mar-Apr, **14(3)**:162-6.

Clatworthy MG, Clark DI, Gray DH, Hardy AE: **Reamed versus unreamed femoral nails. A randomised, prospective trial**. *J Bone Joint Surg Br *1998 May, **80(3)**:485-9.

Cornell CN, Levine D, O'Doherty J, Lyden J: **Unipolar versus bipolar hemiarthroplasty for the treatment of femoral neck fractures in the elderly**. *Clin Orthop Relat Res *1998 Mar, **(348)**:67-71.

David SM, Harrow ME, Peindl RD, Frick SL, Kellam JF: **Comparative biomechanical analysis of supracondylar femur fracture fixation: locked intramedullary nail versus 95-degree angled plate**. *J Orthop Trauma *1997 Jul, **11(5)**:344-50.

Deneka DA, Simonian PT, Stankewich CJ, Eckert D, Chapman JR, Tencer AF: **Biomechanical comparison of internal fixation techniques for the treatment of unstable basicervical femoral neck fractures**. *J Orthop Trauma *1997 Jul, **11(5)**:337-43.

Deshmukh RG, Lou KK, Neo CB, Yew KS, Rozman I, George J: **A technique to obtain correct rotational alignment during closed locked intramedullary nailing of the femur**. *Injury *1998 Apr, **29(3)**:207-10.

Dujardin FH, Benez C, Polle G, Alain J, Biga N, Thornine JM: **Prospective randomized comparison between a dynamic hip screw and a mini-invasive static nail in fractures of the trochanteric area: preliminary results**. *J Orthop Trauma *2001 Aug, **15(6)**:401-6.

Egol KA, Su E, Tejwani NC, Sims SH, Kummer FJ, Koval KJ: **Treatment of complex tibial plateau fractures using the less invasive stabilization system plate: clinical experience and a laboratory comparison with double plating**. *J Trauma *2004 Aug, **57(2)**:340-6.

Gunal I, Taymaz A, Kose N, Gokturk E, Seber S: **Patellectomy with vastus medialis obliquus advancement for comminuted patellar fractures: a prospective randomised trial**. *J Bone Joint Surg Br *1997 Jan, **79(1)**:13-6.

Hahnloser D, Platz A, Amgwerd M, Trentz O: **Internal fixation of distal radius fractures with dorsal dislocation: pi-plate or two 1/4 tube plates? A prospective randomized study**. *J Trauma *1999 Oct, **47(4)**:760-5.

Hardy DC, Descamps, PY, Krallis P, Fabeck L, Smets P, Bertens CL, Delince PE: **Use of an Intramedullary Hip-Screw Compared with a Compression Hip-Screw with a Plate for Intertrochanteric Femoral Fractures. A Prospective, Randomized Study of One Hundred Patients**. *J. Bone Joint Surg. Am *May 1998, **80**:618-30.

Hardy DC, Drossos K: **Slotted intramedullary hip screw nails reduce proximal mechanical unloading**. *Clin Orthop *2003 Jan, **(406)**:176-84.

Hargreaves DG, Drew SJ, Eckersley R: **Kirschner wire pin tract infection rates: a randomized controlled trial between percutaneous and buried wires**. *J Hand Surg Br *2004 Aug, **29(4)**:374-6.

Harrington P, Hihal A, Singhania AK, Howell FR: **Intramedullary hip screw versus sliding hip screw for unstable intertrochanteric femoral fractures in the elderly**. *Injury *2002 Jan, **33(1)**:23-8.

Hofmann GO, Gonschorek O, Buhren V: **Segment transport employing intramedullary devices in tibial bone defects following trauma and infection**. J *Orthop Trauma *1999 Mar-Apr, **13(3)**:170-7.

Horwitz DS, Bachus KN, Craig MA, Peters CL: **A biomechanical analysis of internal fixation of complex tibial plateau fractures**. *J Orthop Trauma *1999 Nov, **13(8)**:545-9.

Janzing HM, Houben BJ, Brandt SE, Chhoeurn V, Lefever S, Broos P, Reynders P, Vanderschot P. **The Gotfried PerCutaneous Compression Plate versus the Dynamic Hip Screw in the treatment of pertrochanteric hip fractures: minimal invasive treatment reduces operative time and postoperative pain***. J Trauma *2002 Feb, **52(2):**293-8.

Jazrawi LM, Kummer FJ, Simon JA, Bai B, Hunt SA, Egol KA, Koval KJ: **New technique for treatment of unstable distal femur fractures by locked double-plating: case report and biomechanical evaluation***. J Trauma *2000 Jan, **48(1)**:87-92.

Jensen CH, Jensen CM: **Biodegradable pins versus Kirschner wires in hand surgery**. *J Hand Surg [Br] *1996 Aug, **21(4)**:507-10.

Jeyam M, Andrew JG, Muir LT, Mcgovern A: **Controlled trial of distal radial fractures treated with a resorbable bone mineral substitute***. J Hand Surg Br *2002 Apr, **27(2)**:146-9.

Johansson T, Jacobsson SA, Ivarsson I, Knutsson A, Wahlstrom O: **Internal fixation versus total hip arthroplasty in the treatment of displaced femoral neck fractures: a prospective randomized study of 100 hips**. *Acta Orthop Scand *2000 Dec, **71(6)**:597-602.

Kankare J, Hirvensalo E, Rokkanen P: **Malleolar fractures in alcoholics treated with biodegradable internal fixation. 6/16 reoperations in a randomized study**. *Acta Orthop Scand *1995 Dec, **66(6)**:524-8.

Karladani AH, Granhed H, Edshage B, Jerre R, Styf J: **Displaced tibial shaft fractures: a prospective randomized study of closed intramedullary nailing versus cast treatment in 53 patients**. *Acta Orthop Scand *2000 Apr, **71(2)**:160-7.

Kligman M, Zecevic M, Roffman M: **The effect of intramedullary corticocancellous bone plug for hip hemiarthroplasty**. *J Trauma *2001 Jul, **51(1)**:84-7.

Koo KH, Kim R, Ko GH, Song HR, Jeong ST, Cho SH: **Preventing collapse in early osteonecrosis of the femoral head. A randomised clinical trial of core decompression**. *J Bone Joint Surg Br *1995 Nov, **77(6)**:870-4.

Kristiansen TK, Ryaby JP, McCabe J, Frey JJ, Roe LR: **Accelerated healing of distal radial fractures with the use of specific, low-intensity ultrasound. A multicenter, prospective, randomized, double-blind, placebo-controlled study**. *J Bone Joint Surg Am *1997 Jul, **79(7):**961-73.

Larsen LB, Madsen JE, Hoiness PR, Ovre S: **Should insertion of intramedullary nails for tibial fractures be with or without reaming? A prospective, randomized study with 3.8 years' follow-up**. *J Orthop Trauma *2004 Mar, **18(3)**:144-9.

Lenzner A, Kaur I, Haviko T, Sogel V, Gapejeva J, Ereline J, Paasuke M: **Impaction bone-grafting increases the holding power of cancellous screws in the femoral head. A pull-out study in human cadaver hips**. *Acta Orthop Scand *1999 Feb, **70(1)**:25-8.

Lunsjo K, Ceder L, Thorngren KG, Skytting B, Tidermark J, Berntson PO, Allvin I, Norberg S, Hjalmars K, Larsson S, Knebel R, Hauggaard A, Stigsson L: **Extramedullary fixation of 569 unstable intertrochanteric fractures: a randomized multicenter trial of the Medoff sliding plate versus three other screw-plate systems**. *Acta Orthop Scand *2001 Apr, **72(2)**:133-40.

Madsen JE, Naess L, Aune AK, Alho A, Ekeland A, Stromsoe K: **Dynamic hip screw with trochanteric stabilizing plate in the treatment of unstable proximal femoral fractures: a comparative study with the Gamma nail and compression hip screw**. *J Orthop Trauma *1998 May, **12(4)**:241-8.

Makwana NK, Bhowal B, Harper WM, Hui AW: **Conservative versus operative treatment for displaced ankle fractures in patients over 55 years of age. A prospective, randomised study**. *J Bone Joint Surg Br *2001 May, **83(4)**:525-9.

McKee MD, Schemitsch EH, Waddell JP, Yoo D: **A prospective, randomized clinical trial comparing tibial nailing using fracture table traction versus manual traction**. *J Orthop Trauma *1999 Sep-Oct, **13(7)**:463-9.

McQueen MM. Redisplaced unstable fractures of the distal radius: **A randomised, prospective study of bridging versus non-bridging external fixation**. *J Bone Joint Surg Br *1998 Jul, **80(4)**:665-9.

Moir JS, Murali SR, Ashcroft GP, Wardlaw D, Matheson AB: **A new functional brace for the treatment of Colles' fractures**. *Injury *1995 Nov, **26(9)**:587-93.

Moroni A, Aspenberg P, Toksvig-Larsen S, Falzarano G, Gianni S: **Enhanced fixation with hydroxyapatite coated pins**. *Clin Orthop Relat Res *1998 Jan, **(346)**:171-7.

Moroni A, Faldini C, Marchetti S, Manca M, Consoli V, Giannini S: **Improvement of the bone-pin interface strength in osteoporotic bone with use of hydroxyapatite-coated tapered external-fixation pins. A prospective, randomized clinical study of wrist fractures***. J Bone Joint Surg Am *2001 May, **83-A(5)**:717-21.

Moroni A, Heikkila J, Magyar G, Toksvig-Larsen S, Giannini S: **Fixation strength and pin tract infection of hydroxyapatite-coated tapered pins**. *Clin Orthop *2001 Jul, **(388)**:209-17.

Nassif JM, Gorczyca JT, Cole JK, Pugh KJ, Pienkowski D: **Effect of acute reamed versus unreamed intramedullary nailing on compartment pressure when treating closed tibial shaft fractures: a randomized prospective study**. *J Orthop Trauma *2000 Nov, **14(8)**:554-8.

Nordeen MH, Lavy CB, Shergill NS, Tuite JD, Jackson AM: **Cyclical micromovement and fracture healing**. *J Bone Joint Surg Br *1995 Jul, **77(4)**:645-8.

Parker MJ, Pryor GA: **Internal fixation or arthroplasty for displaced cervical hip fractures in the elderly: a randomised controlled trial of 208 patients**. *Acta Orthop Scand *2000 Oct, **71(5)**:440-6.

Rodriguez-Merchan EC: **Plaster cast versus percutaneous pin fixation for comminuted fractures of the distal radius in patients between 46 and 65 years of age***. J Orthop Trauma *1997 Apr, **11(3)**:212-7.

Rogmark C, Carlsson A, Johnell O, Sembo I: **A prospective randomised trial of internal fixation versus arthroplasty for displaced fractures of the neck of the femur. Functional outcome for 450 patients at two years***. J Bone Joint Surg Br *2002 Mar, **84(2)**:183-8.

Rogmark C, Carlsson A, Johnell O, Sembo I: **Costs of internal fixation and arthroplasty for displaced femoral neck fractures: a randomized study of 68 patients**. *Acta Orthop Scand *2003 Jun, **74(3)**:293-8.

Sadowski C, Lubbeke A, Saudan M, Riand N, Stern R, Hoffmeyer P: **Treatment of reverse oblique and transverse intertrochanteric fractures with use of an intramedullary nail or a 95 degrees screw-plate: a prospective, randomized study**. *J Bone Joint Surg Am *2002 Mar, **84-A(3):**372-81.

Sanchez-Sotelo J, Munuera L, Madero R: **Treatment of fractures of the distal radius with a remodellable bone cement: a prospective, randomised study using Norian SRS***. J Bone Joint Surg Br *2000 Aug, **82(6)**:856-63.

Schipper IB, Steyerberg EW, Castelein RM, van der Heijden FH, den Hoed PT, Kerver AJ, van Vugt AB: **Treatment of unstable trochanteric fractures. Randomised comparison of the gamma nail and the proximal femoral nail**. *J Bone Joint Surg Br *2004 Jan, **86(1)**:86-94.

Shepherd LE, Shean CJ, Gelalis ID, Lee J, Carter VS: **Prospective randomized study of reamed versus unreamed femoral intramedullary nailing: an assessment of procedures**. *J Orthop Trauma *2001 Jan, **15(1)**:28-32; discussion 32-3.

Stappaerts KH, Deldycke J, Broos PL, Staes FF, Rommens PM, Claes P: **Treatment of unstable peritrochanteric fractures in elderly patients with a compression hip screw or with the Vandeputte (VDP) endoprosthesis: a prospective randomized study**. *J Orthop Trauma *1995, **9(4):**292-7.

Thordarson DB, Samuelson M, Shepherd LE, Merkle PF, Lee J: **Bioabsorbable versus stainless steel screw fixation of the syndesmosis in pronation-lateral rotation ankle fractures: a prospective randomized trial**. *Foot Ankle Int *2001 Apr, **22(4):**335-8.

Tidermark J, Ponzer S, Svensson O, Soderqvist A, Tornkvist H: **Internal fixation compared with total hip replacement for displaced femoral neck fractures in the elderly. A randomised, controlled trial**. *J Bone Joint Surg Br *2003 Apr, **85(3)**:380-8.

Tornetta P 3rd, Tiburzi D: **Reamed versus nonreamed anterograde femoral nailing**. *J Orthop Trauma *2000 Jan, **14(1):**15-9.

Tornetta P 3rd, Tiburzi D: **The treatment of femoral shaft fractures using intramedullary interlocked nails with and without intramedullary reaming: a preliminary report**. *J Orthop Trauma *1997 Feb-Mar, **11(2)**:89-92.

Tropp H, Norlin R: **Ankle performance after ankle fracture: a randomized study of early mobilization**. *Foot Ankle Int *1995 Feb, **16(2)**:79-83.

Tu YK, Lin CH, Su JI, Hsu DT, Chen RJ: **Unreamed interlocking nail versus external fixator for open type III tibia fractures***. J Trauma *1995 Aug, **39(2)**:361-7.

Vossinakis IC, Badras LS: **The external fixator compared with the sliding hip screw for pertrochanteric fractures of the femur**. *J Bone Joint Surg Br *2002 Jan, **84(1)**:23-9.

Waikakul S, Sakkarnkosol S, Vanadurongwan V: **Vascular injuries in compound fractures of the leg with initially adequate circulation**. *J Bone Joint Surg Br *1998 Mar, **80(2):**254-8.

Watson JT, Moed BR, Cramer KE, Karges DE: **Comparison of the compression hip screw with the Medoff sliding plate for intertrochanteric fractures**. *Clin Orthop Relat Res *1998 Mar, **(348):**79-86.

Werber KD, Raeder F, Brauer RB, Weiss S: **External fixation of distal radial fractures: four compared with five pins: a randomized prospective study***. J Bone Joint Surg Am *2003 Apr, **85-A(4):**660-6.

Wheeler DL, Croy TJ, Woll TS, Scott MD, Senft DC, Duwelius PJ: **Comparison of reconstruction nails for high subtrochanteric femur fracture fixation**. *Clin Orthop Relat Res *1997 May, **(338):**231-9.

Wu CC, Chen WJ: **Treatment of femoral shaft aseptic nonunions: comparison between closed and open bone-grafting techniques**. *J Trauma *1997 Jul, **43(1):**112-6.

## Pre-publication history

The pre-publication history for this paper can be accessed here:


